# Association between magnesium oxide use and readmission risk in patients with heart failure and constipation

**DOI:** 10.1186/s40780-025-00478-7

**Published:** 2025-08-08

**Authors:** Junichi Terashima, Takahiro Kambara, Eisei Hori, Risako Koketsu, Teruhiro Sakaguchi, Hiroyuki Osanai, Tomoya Tachi, Tadashi Suzuki

**Affiliations:** 1 Department of Pharmacy, Tosei General Hospital, 160, Nishioiwake-cho, Seto, 489-8642 Aichi Japan; 2 Department of Clinical Pharmacy, Graduate School of Pharmaceutical Sciences, Nagoya City University, 3-1, Tanabedori, Mizuho-ku, Nagoya, 467-8603 Aichi Japan; 3 Department of Cardiovascular Medicine, Tosei General Hospital, 160, Nishioiwake-cho, Seto, 489-8642 Aichi Japan

**Keywords:** Heart failure, Constipation, Readmission, Magnesium oxide, Prognosis

## Abstract

**Background:**

Constipation is frequently observed in patients with chronic heart failure and has been linked to a heightened risk of adverse heart failure outcomes. Although laxatives are commonly prescribed, the optimal choice for individuals with heart failure remains uncertain. We evaluated the association between magnesium oxide use and heart failure prognosis in patients with chronic heart failure.

**Methods:**

A retrospective observational study was conducted on patients admitted to our hospital for heart failure between January 2020 and December 2023 who were continued to be prescribed the same laxatives after discharge. Patients who received magnesium oxide for regular use were categorized into the magnesium oxide group, while all other patients comprised the nonmagnesium oxide group. The primary endpoints were heart failure-related readmission and all-cause mortality, while the secondary endpoint was a composite of both. Propensity scores were calculated based on baseline patient characteristics and used to perform 1:1 nearest-neighbor matching.

**Results:**

During the study period, 171 outpatients with heart failure were prescribed laxatives after hospital discharge, with 74 patients included in the magnesium oxide group. Using propensity score matching, a cohort of 41 matched pairs was established. After matching, the analysis showed that the hazard ratio (HR) for first readmission within 360 d was 0.33 (95% confidence interval [CI]: 0.10–0.92, *p* = 0.035). Additionally, the combined risk of first readmission and all-cause mortality was associated with an HR of 0.30 (95% CI: 0.11–0.82, *p* = 0.019).

**Conclusion:**

Magnesium oxide was strongly associated with a lower risk of readmission and/or death in patients with heart failure who were prescribed laxatives.

**Trial registration:**

N/A. Cases were registered retrospectively.

## Introduction

Constipation is frequently observed in patients with chronic heart failure, and many rely on laxatives to regulate bowel movements. A significant proportion of those with chronic congestive heart failure are older population, a group at higher risk for constipation due to age-related physiological changes [1]. The etiology of constipation in heart failure is multifactorial, including diuretic use and reduced fluid intake, both essential for heart failure management. Additionally, autonomic dysfunction and physical inactivity further impair gastrointestinal motility [1]. Heart failure prognosis is influenced by cardiovascular disease, arrhythmias, diabetes, renal dysfunction, and anemia. Guidelines recommend countermeasures alongside basic drug treatments, such as cardioprotective drugs [2–4]. Despite literature highlighting the association between constipation and cardiovascular risk [5], detailed reports on constipation in patients with heart failure remain limited.

Recent studies indicate that constipation is linked to a higher risk of poor prognosis, including heart failure-related readmission and all-cause mortality [6]. Although chronic heart failure predisposes patients to constipation, the reverse is also true, constipation can worsen heart failure prognosis. Therefore, effective management of bowel function is essential in chronic heart failure.

However, a retrospective study examined the association between constipation and heart failure prognosis in patients with chronic heart failure. It assessed the continued use of laxatives in this population to determine constipation’s prevalence [6–8]. Despite this, methods for managing bowel function remain unaddressed. The efficacy of laxatives in chronic heart failure is yet to be clarified. Additionally, the lack of research comparing specific laxative types and heart failure prognosis highlights the need for comparative studies.

A recent report indicated that magnesium supplementation (magnesium oxide and magnesium sulfate) may reduce mortality risk in patients with severe heart failure with preserved ejection fraction (HFpEF) [9]. According to Japanese guidelines, magnesium oxide is the primary medication recommended for chronic constipation [10]. It has long been used as a laxative and remains widely prescribed due to its affordability. Evaluating the efficacy of affordable and widely available pharmaceutical interventions in chronic heart failure treatment is essential.

We aimed to investigate the relationship between magnesium oxide use as a laxative in patients with chronic heart failure and constipation and heart failure prognosis. The primary endpoints were heart failure-related readmission and all-cause mortality, while the secondary endpoint was a composite of both.

## Methods

### Trial design

This single-center, intergroup comparative retrospective observational study examined electronic medical records of patients hospitalized for heart failure treatment at Tosei General Hospital from January 2020 to December 2023.

The study period considered differences arising from the market introduction and use of newer chronic constipation treatments (lubiprostone, linaclotide, elobixibat, and polyethylene glycol) and advancements in chronic heart failure therapies. This study conformed to the principles outlined in the Declaration of Helsinki and was approved by the Tosei General Hospital Medical Ethics Committee (Approval No.: 1292).

### Study patients

This study included patients hospitalized for heart failure treatment between January 2020 and December 2023 who continued regular outpatient visits for heart failure management and who were continued to be prescribed the same laxatives. We excluded patients who died in the hospital. Prescribed laxatives included magnesium oxide, sennoside, picosulfate, bisacodyl, lubiprostone, linaclotide, elobixibat, polyethylene glycol, and lactulose. Patients were categorized into two groups: The magnesium oxide group, patients prescribed daily magnesium oxide and the nonmagnesium oxide group, with patients prescribed other laxatives, including those using them only as needed.

### Data collection and follow-up

We collected patient characteristics at discharge. Demographic data including age, gender, and body mass index (BMI). Medical history included heart failure history (hospitalization for heart failure, ischemic heart disease, and atrial fibrillation) and diabetes mellitus. Clinical parameters included systolic and diastolic blood pressure, left ventricular ejection fraction (LVEF). Laboratory parameters included albumin, creatinine, blood urea nitrogen (BUN), estimated glomerular filtration rate (eGFR), hemoglobin, brain natriuretic peptide (BNP), and N terminal (NT)-proBNP. We also collected medication data, including antiplatelets, OACs, β-blockers, renin-angiotensin-aldosterone system inhibitors (RAASi), mineralocorticoid receptor antagonists (MRA), sodium-glucose cotransporter-2 inhibitors (SGLT2i), loop diuretics, and irritant laxative (as needed).

Postdischarge data during the observation period were collected from electronic medical records, test results, and referral documents from other hospitals.

### Definition of primary and secondary outcomes

The primary endpoint was first readmission for worsening chronic heart failure after discharge. Secondary endpoints included all-cause mortality and a composite of all-cause mortality and heart failure readmission.

### Statistical analysis

Continuous variables were expressed as mean ± standard deviation (SDs) or median and interquartile range (25th/75th percentile). Categorical variables were reported as frequencies and percentages. Baseline variables were compared using Student’s t-test or Mann–Whitney U test for continuous variables and Fisher’s exact test for categorical variables.

To evaluate the association between first readmission after discharge, all-cause mortality, and regular magnesium oxide use, the Kaplan–Meier method and log-rank test were applied. The Cox proportional hazard model was used to estimate the hazard ratio (HR) and 95% confidence interval (CI). Cases with clear referral to another hospital or consultation intervals of ≥ 3 months were considered censored cases.

To mitigate potential confounders, propensity score matching was applied to ensure comparability between groups.

Seventeen variables influencing heart failure readmission, including age, gender, history of heart failure hospitalization, ischemic heart disease, atrial fibrillation, diabetes, discharge BMI, albumin, BUN, eGFR, hemoglobin, LVEF, and prescriptions of β-blockers, RAASi, MRA, SGLT2i, and irritant laxatives (as needed)—were included in logistic regression analysis to calculate propensity scores (PS). BNP and NT-proBNP were not included as variables; however, the use of other variables related to heart failure risk was considered adequate. Furthermore, we hypothesized that the incorporation of stimulant laxatives as a variable would serve to mitigate the potential confounding effects of concomitant laxative use. The most common missing covariates were BNP levels (*n* = 122, 71.3%) and NT-proBNP levels (*n* = 51, 29.8%), which were not used in the PS calculation. The fully adjusted model was constructed using 1:1 nearest neighbor matching with a caliper width of 0.20 times the SDs of the PS, and no sample replacement was performed. Statistical significance was defined as a two-sided *p*-value < 0.05. All statistical analyses were performed using IBM SPSS Statistics, version 25 (Chicago, IL, USA).

## Results

### Study population

**Fig. 1:**
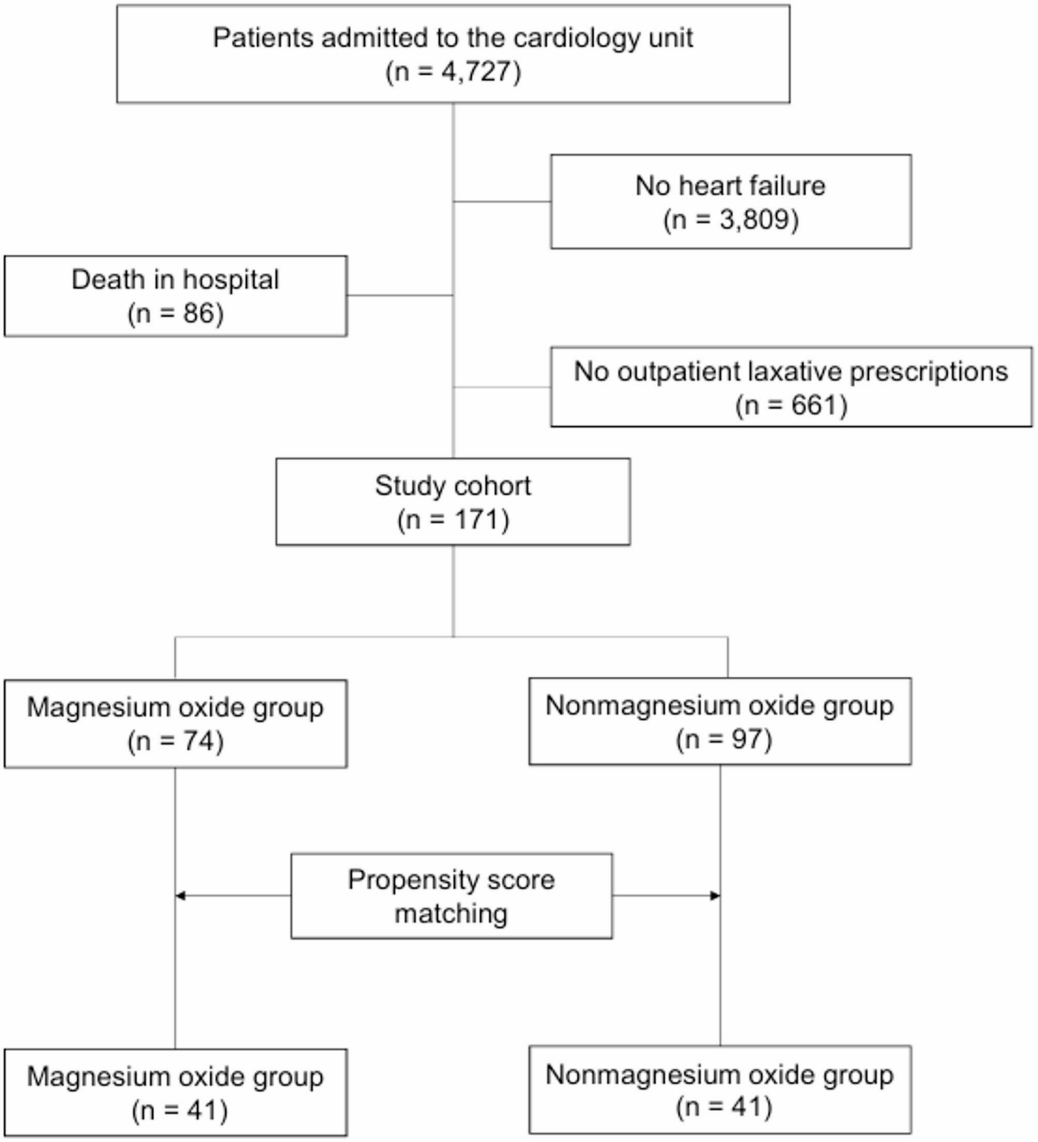
Flowchart illustrating the patient selection process in this study

The patient selection process is illustrated in Fig. 1. A total of 4,727 patients were admitted to the department of cardiology with heart failure between 2020 and 2023. After applying the exclusion criteria, 3,809 patients were excluded on the basis that they did not meet the diagnostic criteria for heart failure. Additionally, 86 were excluded due to death occurring in the hospital. Finally, 661 were excluded because they did not receive continuous outpatient prescription laxative treatment. After excluding ineligible records, the final cohort comprised 171 patients, with 43.3% (74) receiving magnesium oxide therapy. After propensity score matching demonstrated, the matched cohort included 82 patients (41 per group).

### Baseline characteristics

Baseline characteristics were recorded at discharge. The mean age of the 171 patients included in the analysis was 81.0 ± 10.0 years, and 55.6% were male. Most of baseline characteristics of the two groups did not exhibit significant differences. In the nonmagnesium oxide group, patients had higher serum creatinine levels and lower eGFR, and were more frequently prescribed with irritant laxatives as needed (Table 1). Matching improved the balance of these variables. Baseline characteristics are presented in Table 1 (unadjusted model) and Table 2 (fully adjusted model).

**Table 1:** Baseline demographic characteristics

	Overall cohort (*n* = 171)	Magnesium oxide group (*n* = 74)	Nonmagnesium oxide group (*n* = 97)	*p*
Age (years, mean ± SD)	81.0 ± 10.0	81.6 ± 10.3	80.6 ± 9.9	0.314
Male, n (%)	95 (55.6)	39 (52.7)	56 (57.7)	0.516
**Medical history**,** n (%)**				
Previous HF admission	50 (29.2)	19 (25.7)	31 (32.0)	0.370
Ischemic heart disease	80 (46.8)	31 (41.9)	49 (50.5)	0.265
Atrial fibrillation	109 (63.7)	47 (63.5)	62 (63.9)	0.957
Diabetes mellitus	72 (42.1)	33 (44.6)	39 (40.2)	0.568
**Physical examination**				
Systolic blood pressure (mmHg, mean ± SD)	115.9 ± 20.2	115.7 ± 19.1	116.0 ± 21.0	0.785
Diastolic blood pressure (mmHg, mean ± SD)	65.1 ± 11.9	64.4 ± 11.7	65.7 ± 12.2	0.553
Body mass index (kg/m^2^, mean ± SD)	21.7 ± 5.1	20.9 ± 3.6	22.4 ± 5.9	0.220
LVEF (mean ± SD)	0.54 ± 0.14	0.55 ± 0.13	0.53 ± 0.14	0.520
LVEF < 50, n (%)	65 (38.0)	25 (33.8)	40 (41.2)	0.344
Albumin (g/dL, mean ± SD)	3.3 ± 0.5	3.3 ± 0.5	3.3 ± 0.5	0.401
Creatinine (mg/dL, mean ± SD)	1.48 ± 0.86	1.27 ± 0.57	1.64 ± 1.00	0.003
Blood urea nitrogen (mg/dL, mean ± SD)	31.3 ± 17.3	30.6 ± 16.9	32.0 ± 17.6	0.305
eGFR (mL/min/1.73m^2^, mean ± SD)	40.2 ± 19.7	43.9 ± 17.7	37.4 ± 20.7	0.004
Hemoglobin (g/dL, mean ± SD)	11.3 ± 1.9	11.0 ± 1.8	11.6 ± 2.0	0.147
BNP (pg/mL), median (IQR)	458 (294–946)	479 (336–1387)	448 (247–864)	0.196
NT-proBNP (pg/mL), median (IQR)	2,534 (1304–6641)	2,025 (1175–6415)	2,998 (1404–8018)	0.266
**Medication**,** n (%)**				
antiplatelets	56 (32.7)	25 (33.8)	31 (32.0)	0.803
OACs	92 (53.8)	41 (55.4)	51 (52.6)	0.715
Beta-blocker	125 (73.1)	51 (68.9)	74 (76.3)	0.768
ACEi/ARB/ARNI	96 (56.1)	37 (50.0)	59 (60.8)	0.161
MRA	87 (50.9)	35 (47.3)	52 (53.6)	0.417
SGLT2i	43 (25.1)	14 (18.9)	29 (29.9)	0.095
Loop diuretics	146 (85.4)	62 (83.8)	84 (86.6)	0.612
Irritant laxative taken as needed	68 (39.8)	16 (21.6)	52 (53.6)	< 0.0001

Measurements of BNP and NT-proBNP were conducted in 21 and 52 cases within the magnesium oxide group, and in 28 and 68 cases within the nonmagnesium oxide group, respectively

SD, standard deviation; HF, heart failure; LVEF, left ventricular ejection fraction; eGFR, estimated glomerular filtration rate; BNP, brain natriuretic peptide; IQR, Interquartile range; NT-proBNP, N-terminal pro brain natriuretic peptide; OACs, oral anticoagulants; ACEi, angiotensin-converting enzyme inhibitors; ARB, angiotensin receptor blockers; ARNI, angiotensin receptor neprilysin inhibitors; MRA, mineralocorticoid receptor antagonists; SGLT2i, sodium-glucose cotransporter-2 inhibitors

**Table 2:** Baseline demographic characteristics after propensity score matching

	Overall cohort (*n* = 82)	Magnesium oxide group (*n* = 41)	Nonmagnesium oxide group (*n* = 41)	*p*
Age (years, mean ± SD)	81.6 ± 10.3	82.6 ± 9.5	80.6 ± 11.1	0.383
Male, n (%)	47 (57.3)	24 (58.5)	23 (56.1)	1.000
**Medical history**,** n (%)**				
Previous HF admission	22 (26.8)	10 (24.4)	12 (29.3)	0.804
Ischemic heart disease	37 (45.1)	17 (41.5)	20 (48.8)	0.819
Atrial fibrillation	52 (63.4)	27 (65.9)	25 (61.0)	0.957
Diabetes mellitus	31 (37.8)	13 (31.7)	18 (43.9)	0.362
**Physical examination**				
Systolic blood pressure (mmHg, mean ± SD)	115.1 ± 19.5	116.6 ± 19.0	113.6 ± 20.0	0.219
Diastolic blood pressure (mmHg, mean ± SD)	63.9 ± 11.6	64.9 ± 12.1	62.9 ± 11.2	0.472
Body mass index (kg/m^2^, mean ± SD)	21.8 ± 5.7	21.5 ± 3.9	22.1 ± 7.0	0.620
LVEF (mean ± SD)	0.54 ± 0.13	0.54 ± 0.12	0.54 ± 0.14	0.745
LVEF < 50, n (%)	24 (29.3)	11 (26.8)	13 (31.7)	0.809
Albumin (g/dL, mean ± SD)	3.2 ± 0.5	3.2 ± 0.5	3.2 ± 0.5	0.959
Creatinine (mg/dL, mean ± SD)	1.39 ± 0.8	1.32 ± 0.6	1.47 ± 1.0	0.760
Blood urea nitrogen (mg/dL, mean ± SD)	31.4 ± 19.9	28.7 ± 14.5	34.1 ± 24.0	0.487
eGFR (mL/min/1.73m^2^, mean ± SD).	43.5 ± 22.6	43.7 ± 19.3	43.3 ± 25.8	0.467
Hemoglobin (g/dL, mean ± SD)	11.2 ± 1.9	11.3 ± 1.8	11.1 ± 2.0	0.528
BNP (pg/mL), median (IQR)	459 (295–1235)	417 (278–929)	624 (337–1499)	0.259
NT-proBNP (pg/mL), median (IQR)	1,853 (1262–6923)	2,450 (1277–8920)	1,742 (1202–6315)	0.346
**Medication**,** n (%)**				
antiplatelets	27 (32.9)	14 (34.1)	13 (31.7)	1.000
OACs	40 (48.8)	22 (53.7)	18 (43.9)	0.508
Beta-blocker	59 (72.0)	30 (73.2)	29 (70.7)	1.000
ACEi/ARB/ARNI	44 (53.7)	21 (51.2)	23 (56.1)	0.825
MRA	43 (52.4)	19 (46.3)	24 (58.5)	0.377
SGLT2i	19 (23.2)	9 (22.0)	10 (24.4)	1.000
Loop diuretics	71 (86.6)	37 (90.2)	34 (82.9)	0.519
Irritant laxative taken as needed	28 (34.1)	14 (34.1)	14 (34.1)	1.000

SD, standard deviation; HF, heart failure; LVEF, left ventricular ejection Fraction; eGFR, estimated glomerular filtration rate; BNP, brain natriuretic peptide; IQR, Interquartile range; NT-proBNP, N-terminal pro brain natriuretic peptide; OACs, oral anticoagulants; ACEi, angiotensin-converting enzyme inhibitors; ARB, angiotensin receptor blockers; ARNI, angiotensin receptor neprilysin inhibitors; MRA, mineralocorticoid receptor antagonists; SGLT2i, sodium-glucose cotransporter-2 inhibitors

In the entire cohort, missing data were observed only for BNP and NT-proBNP. Measurements of BNP and NT-proBNP were conducted in 21 and 52 cases within the magnesium oxide group, and in 28 and 68 cases within the nonmagnesium oxide group, respectively. No cases were observed where BNP and NT-proBNP were measured concurrently. The reasoning behind this can be elucidated as follows.

The trial period was set to take into account the point at which elobixibat, linaclotide, and lubiprostone had become widely used. Conversely, the elevated rate of missing BNP and NT-proBNP data was associated with the patients’ respective hospitalization periods, which coincided with the period of transition in heart failure biomarker recommendations due to the introduction of angiotensin receptor neprilysin inhibitors.

### Readmission due to heart failure

In the unadjusted model, heart failure readmission within 360 d of discharge occurred in 5 patients (6.8%) in the magnesium oxide group and 20 patients (20.6%) in the nonmagnesium oxide group. Kaplan–Meier analysis showed that time to heart failure readmission within 360 d was significantly longer in the magnesium oxide group (log-rank *p* = 0.023). Similarly, the Cox proportional hazards model indicated a significantly lower risk of heart failure readmission in the magnesium oxide group (HR = 0.34; 95% CI, 0.13–0.90; *p* = 0.030).

**Fig. 2:**
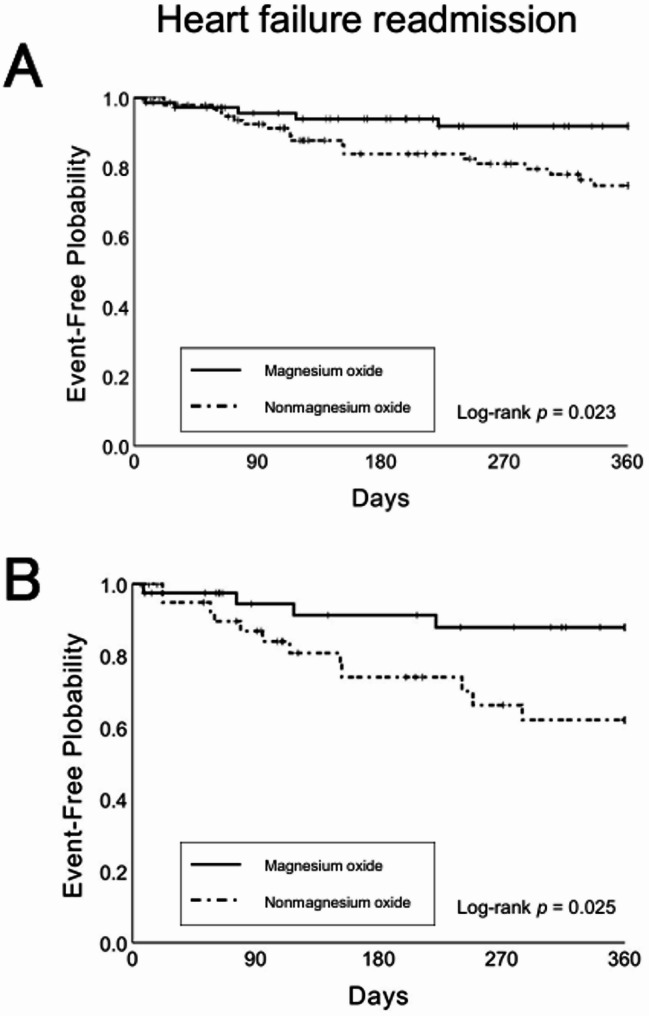
Kaplan–Meier curves for heart failure readmission in the nonmagnesium oxide and magnesium oxide groups, shown before (**A**) and after (**B**) adjustment

In the propensity score-adjusted model, heart failure readmission within 360 d occurred in 4 patients (9.8%) in the magnesium oxide group and 12 patients (29.3%) in the nonmagnesium oxide group. Kaplan–Meier analysis showed that time to heart failure readmission within 360 d was significantly longer in the magnesium oxide group (log-rank *p* = 0.025). Similarly, the Cox proportional hazards model indicated a significantly lower risk of heart failure readmission in the magnesium oxide group (HR = 0.30; 95% CI, 0.10–0.92; *p* = 0.035) (Fig. 2).

### All-cause death

In the unadjusted model, all-cause mortality within 360 d of discharge occurred in 10 patients (13.5%) in the magnesium oxide group and 9 patients (9.4%) in the nonmagnesium oxide group. Kaplan–Meier analysis showed no significant difference in time to all-cause mortality between groups (log-rank *p* = 0.350). Similarly, the Cox proportional hazards model showed no significant difference in all-cause mortality risk (HR = 1.53; 95% CI, 0.62–3.77; *p* = 0.353).

**Fig. 3:**
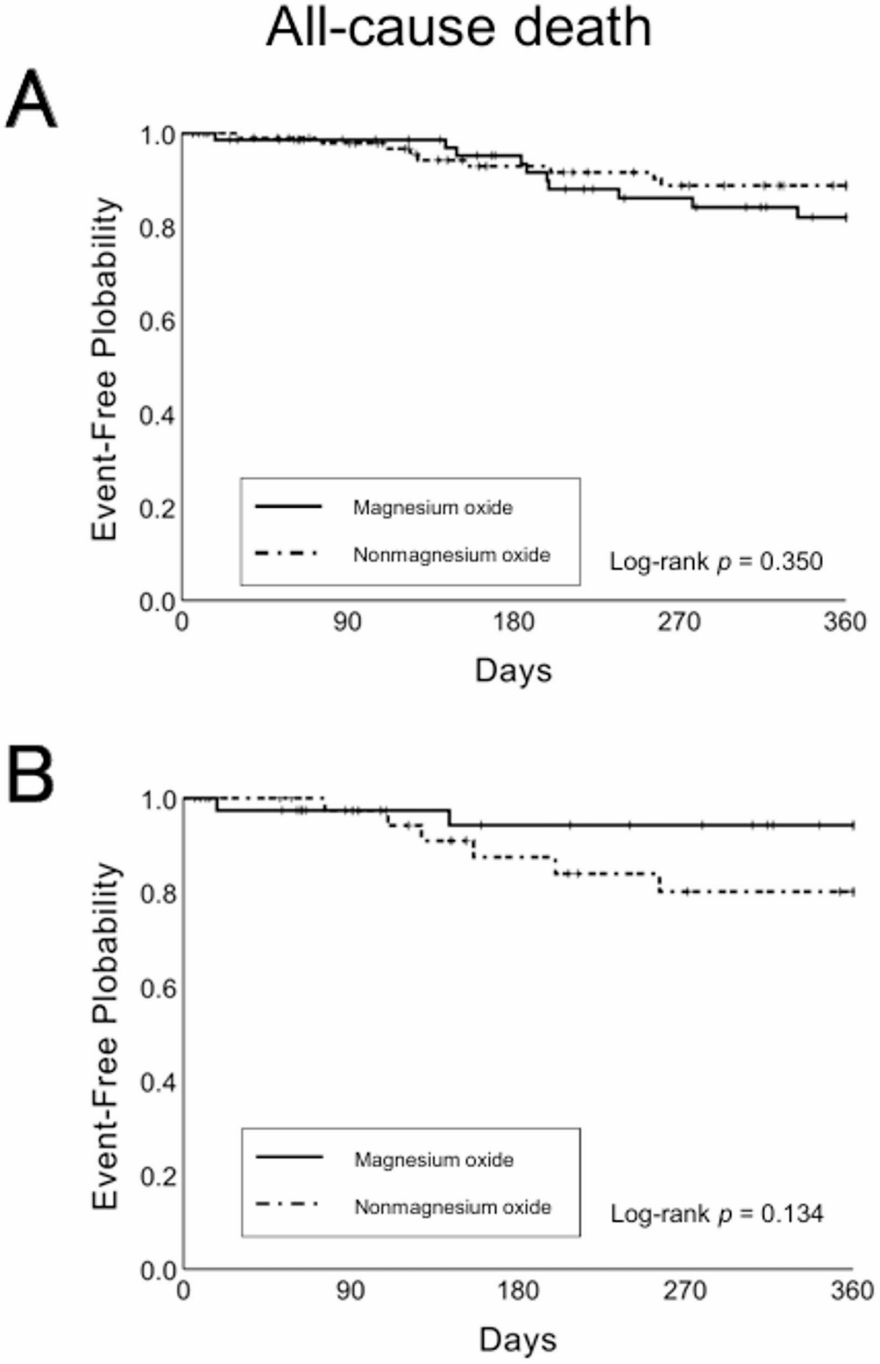
Kaplan–Meier curves for all-cause mortality in the nonmagnesium oxide and magnesium oxide groups, shown before (**A**) and after (**B**) adjustment

In the propensity score-adjusted model, all-cause mortality within 360 d occurred in 2 patients (4.9%) in the magnesium oxide group and 6 patients (14.6%) in the nonmagnesium oxide group. Kaplan–Meier analysis again showed no significant difference in time to all-cause mortality (log-rank *p* = 0.158). Similarly, the Cox proportional hazards model found no significant difference in all-cause mortality risk (HR = 0.313; 95% CI, 0.06–1.56; *p* = 0.156) (Fig. 3).

### Composite endpoint

In the unadjusted model, the composite endpoint within 360 d of discharge occurred in 14 patients (18.9%) in the magnesium oxide group and 25 patients (26.0%) in the nonmagnesium oxide group. Kaplan–Meier analysis showed no significant difference in time to the composite endpoint between groups (log-rank *p* = 0.370). Similarly, the Cox proportional hazards model found no significant difference in composite endpoint risk (HR = 0.74; 95% CI, 0.39–1.42; *p* = 0.372).

**Fig. 4:**
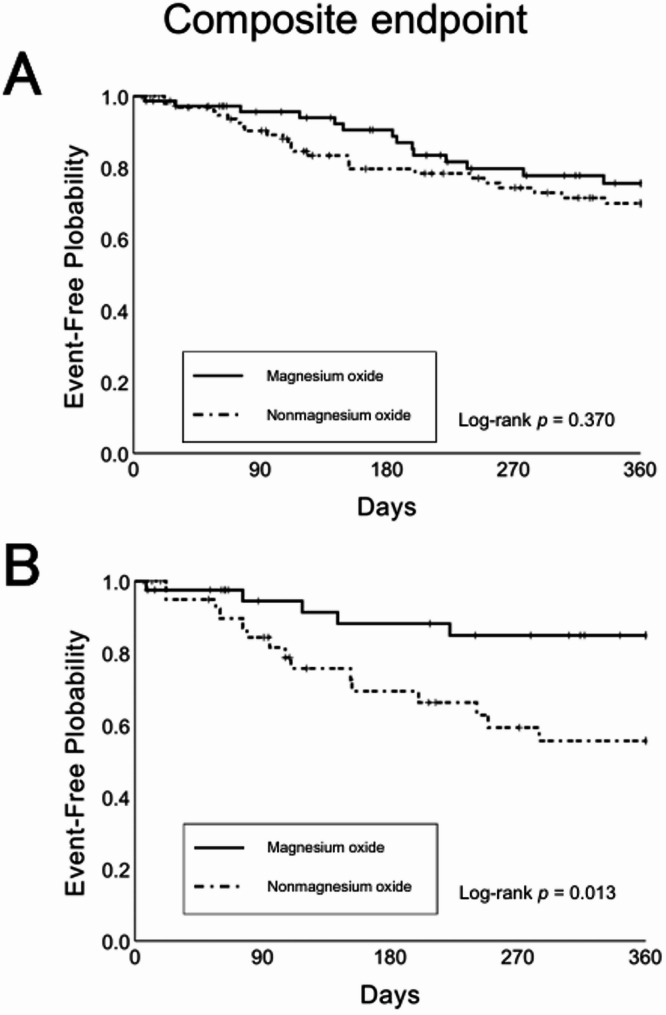
Kaplan–Meier curves for the composite endpoint of all-cause mortality and first heart failure readmission in the nonmagnesium oxide and magnesium oxide groups, shown before (**A**) and after (**B**) adjustment

In the propensity score-adjusted model, the composite endpoint within 360 d of discharge occurred in 5 patients (12.2%) in the magnesium oxide group and 15 patients (36.6%) in the nonmagnesium oxide group. Kaplan–Meier analysis showed that time to the composite endpoint was significantly longer in the magnesium oxide group (log-rank *p* = 0.013). Similarly, the Cox proportional hazards model indicated a significantly lower risk of the composite endpoint in the magnesium oxide group (HR = 0.30; 95% CI, 0.11–0.82; *p* = 0.019) (Fig. 4).

## Discussion

The relationship between constipation and cardiovascular disease has been documented in previous studies [1, 5], and the association between constipation and poor prognosis in heart failure is widely recognized. However, constipation has only recently been directly demonstrated to be an important risk factor for heart failure readmission [6–9]. Moreover, there has been a paucity of interventional studies aimed at mitigating this risk, particularly those focused on drug therapy utilizing laxatives.

This study is distinct from previous studies because it specifically examined patients with heart failure who were prescribed continuous laxative therapy postdischarge and exhibited constipation symptoms. Additionally, it analyzed the various laxative types commonly used for constipation treatment and, to the best of our knowledge, is the first to investigate the suitability of different laxatives in patients with heart failure. Contrary to prior studies that compared patients with heart failure with and without constipation based on laxative use, this study clarifies the impact of laxative therapy on heart failure risk, specifically in patients already receiving regular laxative prescriptions.

Our investigation focused on magnesium oxide, a widely recommended treatment in Japanese guidelines and commonly used for constipation management [10]. Hypomagnesemia is prevalent in heart failure patients, with a multifactorial etiology, including insufficient dietary intake, impaired absorption due to intestinal edema, and increased magnesium excretion from loop diuretics and thiazides [11]. Magnesium plays a role in the electrical and contractile functions of cardiac muscle through the Na+/K ATPase pump, potassium channels, and L-type calcium channels. Therefore, hypomagnesemia can lead to QT prolongation and increase the risk of life-threatening ventricular arrhythmias. Furthermore, impaired regulation of L-type calcium channels and reduced calcium competition may result in increased intracellular calcium levels, further increasing the risk of arrhythmias [11]. Several studies have reported that hypomagnesemia increases the risk of cardiovascular events and worsens heart failure outcomes [12–16]. Evidence suggests that moderate magnesium intake may have cardiovascular benefits and reduce heart failure-related hospitalizations [17–21]. Among the laxatives used in this study (sennoside, picosulfate, bisacodyl, lubiprostone, linaclotide, elobixibat, polyethylene glycol, and lactulose), magnesium supplementation is feasible only with magnesium oxide.

In this study, serum magnesium levels were not adequately measured, limiting the ability to confirm the direct effects of magnesium supplementation. In the included cases, serum Mg levels were obtained during the observation period in 15 cases in the magnesium oxide group and 8 cases in the control group, totaling 23 cases. These evaluations were confirmed using the most recent data collected during the observation period. In the magnesium oxide group, 3 cases exhibited serum Mg levels that exceeded 2.6 mg/dL, indicative of hypermagnesemia. However, no symptoms attributable to this condition were confirmed, and magnesium oxide administration was continued with priority given to managing constipation. Excluding these 3 cases of hypermagnesemia, the mean serum Mg level ± SD was 2.28 ± 0.29 mg/dL in the magnesium oxide group and 2.04 ± 0.27 mg/dL in the control group, with no statistically significant difference between the two (*p* = 0.076). Despite the small number of cases with serum Mg levels available during the observation period, which limited the statistical power, there was a tendency towards higher serum Mg levels in the magnesium oxide group compared to the control group. Magnesium oxide has been shown to increase serum magnesium levels in patients with renal impairment. While hypermagnesemia remains a concern, evidence suggests that magnesium oxide provides substantial benefits in regulating defecation in patients with heart failure.

This study demonstrated that in patients with heart failure and constipation, magnesium oxide therapy was associated with a lower risk of heart failure readmission, and a reduced combined endpoint of heart failure readmission and all-cause mortality. This suggests that magnesium oxide administration in patients with heart failure may reduce the risk of heart failure readmission and lower the combined risk of all-cause mortality and heart failure readmission.

### Study limitations

Although this study accounted for patient background variables, certain limitations must be acknowledged.

Firstly, as this was a single-center retrospective study, it was not possible to verify the balance or impact of unmeasured variables that may have influenced the results.

Secondly, the available data lacked information on functional constipation according to the Rome IV criteria, and on constipation severity, preventing an assessment of its potential effect on heart failure readmission and composite endpoint occurrence.

Further analysis of this study highlighted a limited sample size, which may have resulted in an insufficient number of patients prescribed laxatives other than magnesium oxide.

Additionally, the absence of serum magnesium data makes it difficult to determine the efficacy of magnesium oxide supplementation. Current evidence does not confirm that magnesium oxide intake sufficiently raises magnesium levels to reduce cardiovascular risk.

Future research should include multicenter randomized trials with larger sample sizes, incorporating serum magnesium monitoring and constipation severity assessment as outlined in study protocols.

## Conclusions

In this study, we investigated whether the use of magnesium oxide as a laxative to treat constipation in patients with heart failure differed from the use of other medications. The findings suggest that the utilization of magnesium oxide may play a role in the reduction of heart failure risk.

## Data Availability

No datasets were generated or analysed during the current study.

## Abbreviations

BMI: Body mass index
BNP: Brain natriuretic peptide
BUN: Blood urea nitrogen
CABG: Coronary artery bypass grafting
CI: Confidence intervals
eGFR: Estimated glomerular filtration rate
HFpEF: Heart failure with preserved ejection fraction
HR: Hazard ratio
IQR: Interquartile range
LVEF: Left ventricular ejection fraction
MRA: Mineralocorticoid receptor antagonists
NT-proBNP: N-terminal pro-B-type natriuretic peptide
PCI: Percutaneous coronary intervention
PS: Propensity scores
RAASi: Renin-angiotensin-aldosterone system inhibitors
SD: Standard deviation
SGLT2i: Sodium-glucose cotransporter-2 inhibitors
